# Neural Blockade Anaesthesia of the Mandibular Nerve and Its Terminal Branches: Rationale for Different Anaesthetic Techniques Including Their Advantages and Disadvantages

**DOI:** 10.1155/2011/307423

**Published:** 2011-05-25

**Authors:** Jason Khoury, Grant Townsend

**Affiliations:** School of Dentistry, The University of Adelaide, Adelaide, SA 5005, Australia

## Abstract

Anaesthesia of structures innervated by the mandibular nerve is necessary to provide adequate pain control when performing dental and localised surgical procedures. To date, numerous techniques have been described and, although many of these methods are not used routinely, there are some situations where their application may be indicated. Patient factors as well as anatomical variability of the mandibular nerve and associated structures dictate that no one technique can be universally applied with a 100% success rate. This fact has led to a proliferation of alternative techniques that have appeared in the literature. This selective review of the literature provides a brief description of the different techniques available to the clinician as well as the underlying anatomy which is fundamental to successfully anaesthetising the mandibular nerve.

## 1. Introduction

Neural blockade anaesthesia is necessary for anaesthesia of much of the mandibular bone as well as lower posterior teeth, which cannot be readily anaesthetised via supraperiosteal deposition of local anaesthetic [1]. Although there is considerable anatomical variation, portions of the mandible consist of dense, thickened bone, making it difficult for externally deposited local anaesthetic to diffuse toward the inferior alveolar nerve (IAN) that lies within the substance of the mandible. Hence, clinicians commonly attempt to anaesthetise this nerve, which supplies all mandibular teeth on the ipsilateral side, before it enters the mandibular canal via blockade anaesthesia. Considering that the peripheral extension of the mandibular nerve, after it leaves the cranial base, is not encased in bone for some distance, there are opportunities to administer blockade anaesthesia at multiple levels. Although many techniques for mandibular blockade anaesthesia are practised, the direct inferior alveolar nerve block (IANB) [2], the indirect IANB [3], the Akinosi closed-mouth technique [4], the Gow-Gates technique [5], and variations thereof are most commonly used internationally, and this paper will focus on these approaches. Some extraoral techniques via the mandibular notch, which are useful in some trauma patients [6], and a recently described intraoral anterior injection technique, a variation of the indirect IANB technique, are also described [7].

## 2. Anatomy

A thorough knowledge of anatomy is crucial in providing predictable, safe, and effective mandibular anaesthesia. The mandibular nerve is the largest branch of the trigeminal nerve's three main branches which separate at the trigeminal ganglion near the cavernous sinus. It passes through foramen ovale and descends into the infratemporal region for a short distance as a single trunk before dividing into anterior and posterior branches that pass down into the pterygo-mandibular space. The anterior branch is mostly involved with supplying motor innervation to muscles of mastication, while the posterior branch is predominantly associated with sensory function to the tongue, lower gingivae, mandibular bone, teeth, and part of the lower lip and chin on the ipsilateral side. The mandibular nerve also provides a route by which post-ganglionic parasympathetic facial nerve fibers can travel to the structures that they supply, such as salivary glands.

**Figure 1:**
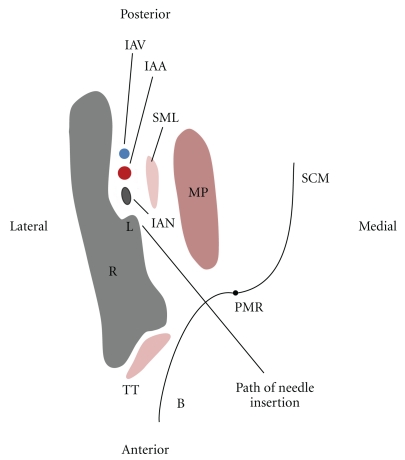
Transverse section of the mandibular ramus at the level just superior to the lingula. (R: Ramus, IAN: inferior alveolar nerve, IAV: inferior alveolar vein, IAA: inferior alveolar artery, SML: sphenomandibular ligament, MP: medial pterygoid muscle, B: buccinator, PMR: pterygomandibular raphe, SCM: superior constrictor muscle, TT: tendon of temporalis L: lingula). The needle is shown passing through the pterygomandibular space where it is directed to an area of bone just superior to the lingula, L. This is the level at which an IANB should be administered.

The main objective of mandibular blockade anaesthesia is to anaesthetise the posterior branch of the mandibular nerve and its distal branches. The extraosseous course of the mandibular nerve is predominantly within the pterygomandibular space which is a small fascial-lined cleft containing mostly loose areolar tissue (Figure 1) [8]. It is bounded medially and inferiorly by the medial pterygoid muscle and laterally by the medial surface of the mandibular ramus [9]. Posteriorly, parotid glandular tissue curves medially around the back of the mandibular ramus to form a posterior border, while, anteriorly, the buccinator and superior constrictor muscles come together to form a fibrous junction, the pterygomandibular raphe (Figure 1). Most mandibular block procedures involve deposition of local anaesthetic solution within the pterygomandibular space via an intraoral route, namely, by piercing the buccinator muscle anteriorly.

The posterior division of the mandibular nerve gives rise to three nerves as it descends: the lingual, inferior alveolar, and auriculotemporal nerves. Neural blockade anaesthesia of the mandibular nerve may stop pain conduction for any number of these nerves depending on the amount of anaesthetic solution administered, the location in the horizontal plane of drug administration, as well as the height in the vertical plane at which the block is given. All of these nerves have important roles in innervating oral and temporomandibular structures, and anaesthetising them is advantageous before carrying out potentially painful surgical or dental procedures. 

In addition to the neural aspects of the pterygomandibular space, there are vascular pathways, fibrous tissue elements, muscular structures, and glandular tissue that need to be considered to improve the predictability, effectiveness, and safety of blockade anaesthesia. Vessels such as the inferior alveolar, maxillary, and external carotid arteries are present within the vicinity of many mandibular block procedures, and damage to these structures may lead to a variety of complications. Similarly, venous components of the vasculature such as the inferior alveolar, retromandibular, and maxillary veins, as well as elements of the pterygoid venous plexus, are also within close proximity to the areas where many mandibular block injections are administered and may be inadvertently damaged, especially if techniques are careless or inappropriate. 

Fibrous tissue elements such as the sphenomandibular ligament and interpterygoid fascia have a bearing on diffusion dynamics within the anatomical spaces relevant to mandibular block procedures [10]. In vivo studies involving the radiographic analysis of injected local anaesthetics mixed with contrasting medium have found that local anaesthetic solution diffuses easily through the loose areolar tissue that is present in many anatomical spaces [7, 11]. However, deposition of local anaesthetic in a location where it is separated from its intended target by fibrous tissue may impede its diffusion [10, 12]. This is especially relevant for mandibular block techniques that are administered at a low level, as fibrous tissue converges inferiorly onto the bone, restricting the available area where the needle tip can be placed and not be in a position where it is separated from the intended target (i.e., IAN) by fibrous tissue. 

The pterygoid muscles occupy considerable space within the area where needles are directed during mandibular block procedures. The medial pterygoid muscle lies in a position where, if needle placement is too medial during a low block technique such as the direct or indirect IANB, this may result in intramuscular injection, potentially leading to less effective anaesthesia and postoperative trismus [1]. Similarly, for high block techniques such as the Gow-Gates and Akinosi closed mouth techniques, needle placement that is too medial may likewise result in local anaesthetic deposition intramuscularly, within the substance of the lateral pterygoid muscle.

## 3. Techniques

### 3.1. Direct IANB

**Figure 2:**
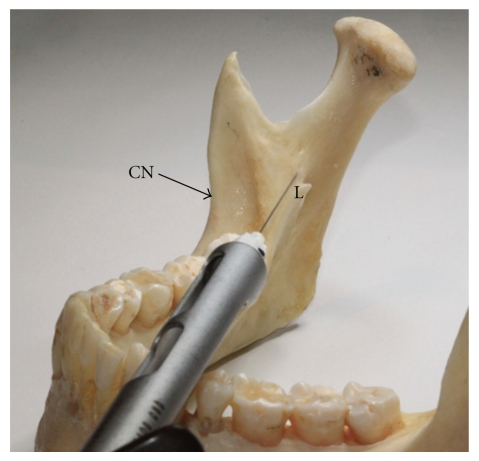
Photograph of the mandible where the needle tip is directed toward the area of bone just superior to the lingula. This positioning of the needle will allow for local anaesthetic deposition in a location in close proximity to the IAN and associated vessels, yet minimising the risk of damaging them. This photograph reflects where local anaesthetic is injected with the direct and indirect IANB (CN: Coronoid notch, L: Lingula).

Although there are many techniques described, the direct IANB, also known as the direct thrust approach, remains one of the most commonly used to obtain mandibular anaesthesia [13]. The direct IANB technique involves needle insertion into the pterygomandibular space by piercing the buccinator muscle, anteriorly. Once in this anatomical space, the objective of this technique is to deposit local anaesthetic solution near the inferior alveolar nerve, just before it enters the mandibular foramen that leads into the mandibular canal. Although the exact location of where the needle tip should be located in relation to the IAN is hard to assess in any one patient due to the required ~20–25 mm depth of tissue penetration [14], it is advantageous to administer the injection so that the tip of the needle contacts bone just superior to the tip of the lingula (Figure 2). This will ensure that local anaesthetic solution is not deposited medial to the sphenomandibular ligament. The lingual nerve lies medial and anterior to the IAN and can be anaesthetised during an IANB by withdrawing the needle and swinging the barrel of the syringe toward the dental midline.

**Figure 3:**
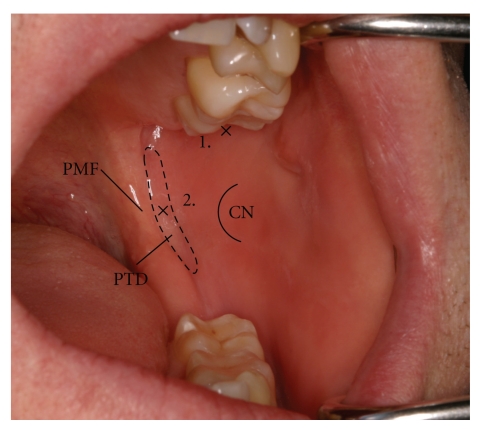
Intraoral photograph of the left side of the oral cavity showing the injection sites for different mandibular block techniques. The pterygotemporal depression exists between the pterygomandibular fold and coronoid notch and represents the area where a direct or indirect IANB is administered in the mediolateral plane. The height at which this block is given is approximately the level of the coronoid notch. In contrast, the Gow-Gates mandibular block is administered at a much higher level. The mesiopalatal cusp of the upper second molar determines the height of the injection while the site in the mediolateral plane is the area of tissue just posterior to the upper second or third molar (PTD: pterygotemporal depression, PMF: pterygomandibular fold, CN: coronoid notch, 1: area where a direct/indirect IANB would be administered, 2: area where a Gow-Gates mandibular block would be administered).

Clinicians use several intraoral landmarks when administering direct IANBs. When the mouth is wide open, the pterygotemporal depression represents the site of injection and is positioned between the raised ridge of mucosa overlying the pterygomandibular raphe medially and the mucosa that overlies the anterior border of the mandibular ramus and tendon of temporalis laterally (Figure 3). The visible intraoral ridge produced by the underlying pterygomandibular raphe is referred to as the pterygomandibular fold (Figure 3).

 The level of injection is established by the point of maximum concavity on the anterior border of the mandibular ramus, an area referred to as the coronoid notch. Alternate landmarks for assessing the correct height of needle entry for the IANB includes inserting the needle approximately 10 mm above the lower occlusal plane when the mouth is fully open [1]. Other guidelines include inserting the needle at the midway point between the upper and lower posterior teeth when the mouth is wide open and visualising the apex of the buccal pad of fibrous tissue that forms an apex close to the pterygomandibular fold [10]. The buccal pad is a submucosal fibrous band of tissue separating the overlying mucosa from the underlying buccinator muscle, and it should not be confused with the buccal pad of fat which is an area of adipose tissue between the buccinator muscle and masseter muscle. 

The required horizontal angulation of the syringe varies between patients with the morphology of the internal oblique ridge, degree of ramal flaring, shape of the lingula, dental arch type, and alignment of teeth influencing what is appropriate. As a guide, the syringe barrel should extend over the premolars on the contralateral side [1]. This angulation, however, may need to be modified if the needle tip has not made bone contact at the appropriate depth of around 20–25 mm [14]. When the correct depth of needle penetration and angulation has been attained, the needle is then withdrawn one to two millimetres and an aspiration test performed. 

Some authors have emphasised the importance of extraoral landmarks in addition to intraoral landmarks to evaluate ideal needle placement and angulation, such as the degree of ramal flaring and the height and width of the mandibular ramus [8]. This is especially important with the edentulous patient.

Considering the height at which the direct IANB is administered, it only anaesthetises the IAN, LN, and nerve to mylohyoid, in most cases. Generally, anaesthesia of these nerves is all that is required for most dental and local surgical procedures. If, however, other nerves require anaesthesia, or if anatomical variation in an individual results in a disruption in the usual pattern of nerves that a low nerve block would normally anaesthetise, then a higher nerve block technique may be indicated.

### 3.2. Indirect IANB

The indirect IANB is a variation of the direct technique where the level and site of injection is the same. The fundamental difference, however, lies in the method by which the appropriate depth of needle insertion (~20–25 mm) is obtained. The indirect technique involves the insertion of the needle using the same landmarks to indicate correct height and mediolateral needle placement (Figure 3), but with a significantly greater degree of syringe angulation on the contralateral side [3]. The effect of this is to make early bone contact near the anterior border of the ramus, anterior to the mandibular foramen. Subsequent to this, the needle angulation is slowly altered by swinging the barrel of the syringe toward the midline, thus allowing the needle to penetrate to progressively deeper positions through soft tissue. This process is continued until the appropriate depth of needle insertion (~20–25 mm) is attained [3].

The level at which the indirect IANB is administered is the same as the direct IANB, and, consequently, it has the same advantages and limitations. In addition to this, it is worth noting that the degree of tissue damage sustained to the contents of the pterygomandibular space may be greater than the direct IANB due to the movement of the partially inserted needle as the barrel of the syringe is swung toward the midline.

### 3.3. Anterior Injection Technique

**Figure 4:**
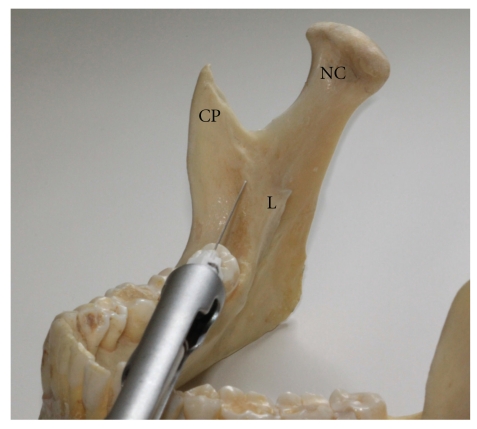
Photograph of the mandible showing the positioning of the needle tip when administering the anterior IANB. Note that the location of the needle tip is a considerable distance from where the IAN would be expected. This technique relies heavily on the ability of local anaesthetic to diffuse throughout the pterygomandibular space (NC: neck of Condyle, CP: coronoid process, L: lingula).

The anterior injection technique involves the insertion of the needle for approximately 10 mm into the pterygomandibular space where the local anaesthetic is deposited, allowing for the anaesthetic solution to slowly diffuse across the space and toward the IAN [7]. Firstly, the needle insertion site is determined using the same anatomical landmarks as for the direct and indirect IANB (Figure 3). The anterior injection technique also requires a large degree of horizontal needle angulation, similar to the indirect IANB, where the barrel of the syringe lies over the contralateral molars. The fundamental difference between the techniques, however, is that when initial bone contact is made with the needle at ~10 mm (Figure 4), no further insertion of the needle into the pterygomandibular space is attempted. The suggested advantage of this technique over the others is that the risk of vascular and neural damage is said to be reduced as the needle does not penetrate as deeply [7].

### 3.4. Gow-Gates Mandibular Block Technique

**Figure 5:**
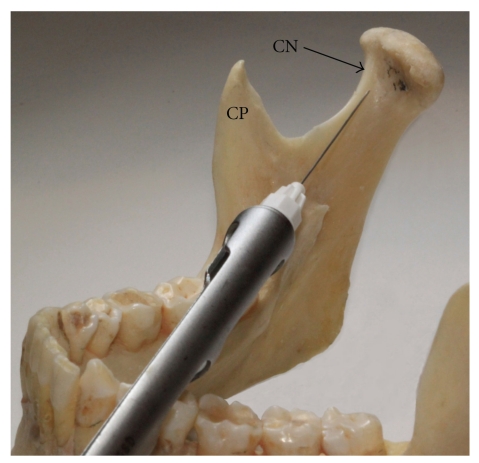
Photograph of the mandible showing the ideal needle tip position when administering the Gow-Gates mandibular block technique. The intended target area for the needle is the lateral condylar neck region, below the insertion of the lateral pterygoid muscle and the attachment of ligaments associated with the temporomandibular capsule (CN: condylar neck, CP: coronoid process).

The Gow-Gates mandibular block is often referred to as a true mandibular block as the distribution of its effect is larger than that of lower-level nerve block techniques and it anaesthetises the auriculotemporal and long buccal nerves in most cases [1]. This technique involves the intraoral insertion of a needle through the pterygomandibular space until bony contact is made with the anterolateral condylar neck, under the insertion of the lateral pterygoid muscle, in an area referred to as the processus condyloideus [15] (Figure 5). Although different for every patient, the average depth of needle insertion is ~25 mm, and following needle contact, withdrawal of 1-2 mm, and an aspiration test is required [16]. In addition to this, Malamed has suggested that the patient should keep their mouth open for ~60–90 seconds following injection to allow for more speedy diffusion of local anaesthetic as mandibular opening reduces the distance between where the local anaesthetic is deposited and the mandibular nerve [1].

A combination of intraoral and extraoral landmarks are used for the Gow-Gates mandibular block technique. Firstly, the height of injection is established by the mesiopalatal cusp of the maxillary second molar [1]. Secondly, the site of injection is the tissue immediately distal to the maxillary second molar (or maxillary third molar if present) [1]. The angulation of the syringe in the mediolateral plate involves the barrel of the syringe being approximately in the corner of the mouth, over the contralateral premolars while the orientation of the syringe in the vertical plane requires the alignment of the syringe with the plane that extends between the lower border of the tragus, in the intertragic notch, and the corner of the mouth [17]. As with other techniques, the underlying anatomy may differ between individuals, and due to the significant depth of tissue penetration (~25 mm) required, it is difficult to assess the specific location of the needle tip in any one individual. 

The simultaneous visualisation of intraoral and extraoral landmarks required when administering the Gow-Gates mandibular block can be difficult and is often listed by clinicians as a reason why they prefer other mandibular block techniques [13]. Clinical experience with the technique, however, is considered to overcome early difficulties that may be faced when first applying the technique [1]. In addition to this, the time required for onset of anaesthesia is greater than that for the direct IANB due to the greater distance between the site where local anaesthetic is deposited and the mandibular nerve (~5–10 mm), as well as the larger size of the nerve trunk at this relatively higher level [1]. The level at which the injection is given, however, has the advantage of anesthetising more terminal branches of the mandibular nerve than lower-level block techniques, reducing the need for additional injections to supplement the initial block.

### 3.5. Akinosi Closed-Mouth Technique

The Akinosi closed-mouth mandibular block approach provides an alternate technique for individuals who have limited mouth opening, which is a distinct contraindication for the other block techniques [1, 4]. This technique involves the intraoral insertion of a needle into the pterygomandibular space for ~25–30 mm [18] while the mouth is fully closed. This technique does not involve bony contact, where the desired location of the needle tip should be in the loose areolar tissue medial to the mandibular ramus. As with other block procedures, an aspiration test is performed before deposition of local anaesthetic [18].

The site of injection for this technique is at the level of the mucogingival junction of the maxillary second or third molar [1]. This places the syringe deep in the buccal fornix where the barrel of the syringe needs to be parallel to the maxillary dentition. Ramal flaring should be gauged extraorally, and the path of needle insertion should be parallel to this.

**Figure 6:**
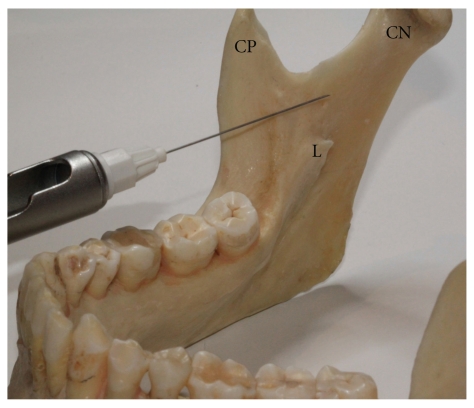
Photograph of the mandibular ramus from a medial view showing the needle tip positioning required for the Akinosi closed mouth mandibular nerve block technique. Note that the needle should not contact bone during needle insertion. The needle tip slips along the medial aspect of the ramus to its intended target area, the loose areolar tissue within the superior reach of the pterygomadibular space (CN: condylar neck, CP: coronoid process, L: lingula).

Considering the high level at which this block is administered, it shares the advantages of other high level blocks in the distribution of its anaesthetic effect. Although this technique can be used for anyone, most clinicians reserve it for those who have severe mouth opening deficiencies or are severe gaggers [19]. As no bony contact is made with any structure (Figure 6), the uncertainty surrounding the localisation of the needle tip when the needle is fully inserted in the tissue is a unique disadvantage of this technique [1].

## 4. Supplementation to Blockade Anaesthesia with Local Supraperiosteal Infiltrations

The success rates of some injection techniques can be quite low, especially with direct and indirect IANBs where the rate of success can be as low as 80% [1]. As a result, supplementation of these injections with local infiltrations has been suggested, and clinical trials have been performed to evaluate their effectiveness. These infiltration injections should not be confused with buccal nerve blocks or infiltrations which are administered following a direct or indirect IANB to anaesthetise the long buccal nerve which innervates the buccal gingivae of lower posterior molars. For example, the long buccal nerve and the lingual nerve must be anaesthetised, in addition to the IAN, if lower posterior teeth are to be extracted. 

There are many reasons why anaesthetic failures occur, and they can often be classified into two major categories: poor operator technique and anatomical variation [20]. As it is difficult to determine the anaesthetic outcome in any given patient, the use of supplementary infiltrations from the outset has been suggested as a safety net for individuals who respond less favourably to IANBs. Most in vivo research demonstrates an increased effectiveness of anaesthesia with supplementary infiltrations [21–23]; however, Foster et al. [24] were not able to observe a statistically significant difference. This may be explained by the type of local anaesthetic formulation used. Foster et al. used 2% Lidocaine HCL with 1 : 100000 adrenaline, and Hasse et al. [22] observed that this preparation did not produce a statistically significant additional effect to mandibular anaesthesia when used as a supplementary infiltration following an IANB, in contrast to the use of 4% Articaine HCL with 1 : 100000 adrenaline as tested in the same research project. Considering that buccal and lingual infiltrations alone when administered to lower first molars in the absence of blockade anaesthesia produce anaesthesia in about 38.7 to 64.5% of individuals with the use of Lidocaine HCL and Articaine HCL, respectively [25], it is likely that supplementary injections will add to the anaesthetic effectiveness of an IANB. However, much more research needs to be conducted in this area to further our understanding of the best approaches to mandibular anaesthesia.

The results of studies used to compare the efficacy of different local anaesthetic agents when given as supplementary infiltrations can be difficult to evaluate due to the different formulation concentrations used, the different volumes injected, the different criteria used for describing profound anaesthesia, and other differences in the research methodology (i.e., which teeth are tested). Most studies indicate that Articaine HCL is more effective than Lidocaine HCL when administered as a supplementary infiltration. However, the concentration of the active ingredient in the former is double that of the latter, 4% and 2%, respectively, making comparisons difficult. More research needs to be conducted to evaluate whether the type of anaesthetic agent used has an impact on the extent and level of anaesthesia attained.

## 5. Conclusion

Mandibular anaesthesia is an essential part of clinical practice for dental and local surgical procedures in the oral region. Due to various factors, anaesthesia of the mandibular nerve is associated with a high degree of failure, especially with the use of low-level block procedures. Failure of anaesthesia can prove challenging for the clinician to understand. If a mandibular block procedure fails, it is essential that the operator carefully evaluates his/her technique, and considers common anatomical variations, to determine what may have contributed to the problem. If the underlying cause is likely to be due to anatomical variation, then the use of an alternative method is indicated rather than repetition of the same technique.
